# In Vitro Antineoplastic Effects of *Zataria multiflora*: A Systematic Review of Mechanisms, Selectivity, and Synergy

**DOI:** 10.1002/fsn3.72050

**Published:** 2026-06-30

**Authors:** Alireza Khanahmad, Fatemeh Peymaninezhad, Fariba Sharififar, Ali Sadatmoosavi, Ali Bazi, Roohollah Mirzaee Khalilabadi, Hajar Mardani Valandani

**Affiliations:** ^1^ Student Research Committee Kerman University of Medical Sciences Kerman Iran; ^2^ Department of Hematology and Medical Laboratory Sciences, Faculty of Allied Medicine Kerman University of Medical Sciences Kerman Iran; ^3^ Shahreza Health Network Isfahan University of Medical Sciences Isfahan Iran; ^4^ Stem Cells and Regenerative Medicine Innovation Center Kerman University of Medical Sciences Kerman Iran; ^5^ Herbal and Traditional Medicines Research Center Kerman University of Medical Sciences Kerman Iran; ^6^ Department of Pharmacognosy, Faculty of Pharmacy Kerman University of Medical Sciences Kerman Iran; ^7^ Neuroscience Research Center, Institute of Neuropharmacology Kerman University of Medical Sciences Kerman Iran; ^8^ Faculty of Allied Medical Sciences Zabol University of Medical Sciences Zabol Iran

**Keywords:** complementary therapies, phytogenic antineoplastic agents, plant extracts, systematic review, *Zataria multiflora*

## Abstract

*Zataria multiflora* (ZM), a plant with recognized ethnomedicinal properties, harbors a spectrum of bioactive compounds demonstrating cytotoxic potential against various cancer cell lines. However, the absence of a systematic synthesis of individual research efforts has significantly impeded the translation of promising in vitro findings into rigorous clinical investigations. This study aims to systematically review and synthesize the current evidence regarding the in vitro anticancer effects of ZM. A meticulous systematic search, adhering to the PRISMA guidelines, was executed on September 17, 2025, across major scientific databases, including Web of Science, Medline, Scopus, Embase, Magiran, and Google Scholar. The search strategy incorporated relevant keywords, Medical Subject Headings (MeSH) terms, and their Persian equivalents to ensure comprehensive coverage. Retrieved articles underwent rigorous screening to identify eligible studies, and literature saturation was confirmed through a manual review of the reference lists of the selected publications. From an initial pool of 107 articles retrieved from databases as well as 52 articles identified through manual searching, a total of 29 papers were included. We reviewed the cytotoxicity and safety of ZM across 24 cancer cell lines (derived from 13 neoplastic diseases) and six normal cell types. While 17 studies explored the anticancer effects of ZM, 8 investigated its nanoscale formulations, and 4 combined both methods. ZM consistently demonstrated significant pro‐apoptotic effects in cancer cell lines, a phenomenon not observed in normal cells, highlighting its selective cytotoxicity. Previous studies have also reported synergistic effects between ZM and conventional anticancer agents, including doxorubicin, as well as with radiotherapy. A prominent mechanism of action identified was cell cycle arrest (in G1, G2, or S phases) in cancer cells. Preliminary in vitro evidence suggests the promising role of ZM in inducing apoptosis, prompting cell cycle arrest, and reducing proliferation in various cancer cell models. However, to conclusively establish the efficacy and safety of ZM in clinical settings, further research employing advanced models, such as spheroid‐based cell cultures, in vivo animal models, and well‐designed clinical trials, is indispensable.

## Graphical abstract

*Zataria multiflora* (ZM) is an aromatic plant containing various bioactive components, including phenolic compounds, flavonoids, and terpenoids. Here, we systematically review 27 articles to explore the effect of ZM on different cancer/normal cell lines. Both ZM and its nanoforms demonstrated selective cytotoxicity and apoptosis induction toward cancer cells. They also showed a synergistic effect when combined with traditional anticancer agents.

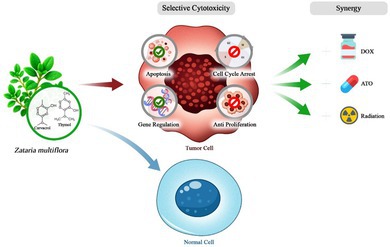

## 1 Introduction

Cancer is a major public health issue in the modern era, accounting for almost 20 million new cases and 9.7 million (16.8% of) deaths worldwide in 2022. It is estimated that one in five human beings develops cancer during their lifespan (Bray et al. 2024). Despite recent advancements in traditional and novel anticancer therapeutic agents, concerning limitations, including off‐target effects, adverse events, toxicity induction, high costs, low biocompatibility, and variable efficacy and safety persist (Farrokhi et al. 2025; Khazaee‐Nasirabadi et al. 2024; Joshi et al. 2024; Liu et al. 2024). Therefore, natural compounds and phytochemicals have gained interest.

The history of using medicinal plants dates back to ancient times, with Ayurveda, traditional Indian medicine, and traditional Chinese medicine being among the oldest. Natural products have played a pivotal role in anticancer drug discovery. Between 1940 and 2014, nearly half of all approved anticancer medications were either natural products or their derivatives. Prominent examples include Vinca alkaloids (e.g., vincristine), taxanes (e.g., paclitaxel, docetaxel), and podophyllotoxins (e.g., etoposide) (Choudhari et al. 2019). Since botanical derivatives contain various novel bioactive compounds, they can offer diverse anticancer mechanisms (Banerjee et al. 2023; Zhang et al. 2022). Additionally, plant‐based approaches often exhibit lower toxicity rates and help overcome drug resistance, either as a standalone approach or in combination with existing therapeutic agents (Zhang et al. 2022; Khanahmad et al. 2024). Overall, scientific validation of traditional medicine bridges ancient and modern medical knowledge, leading to the development of safer and more effective cancer therapies.

**FIGURE 1:**
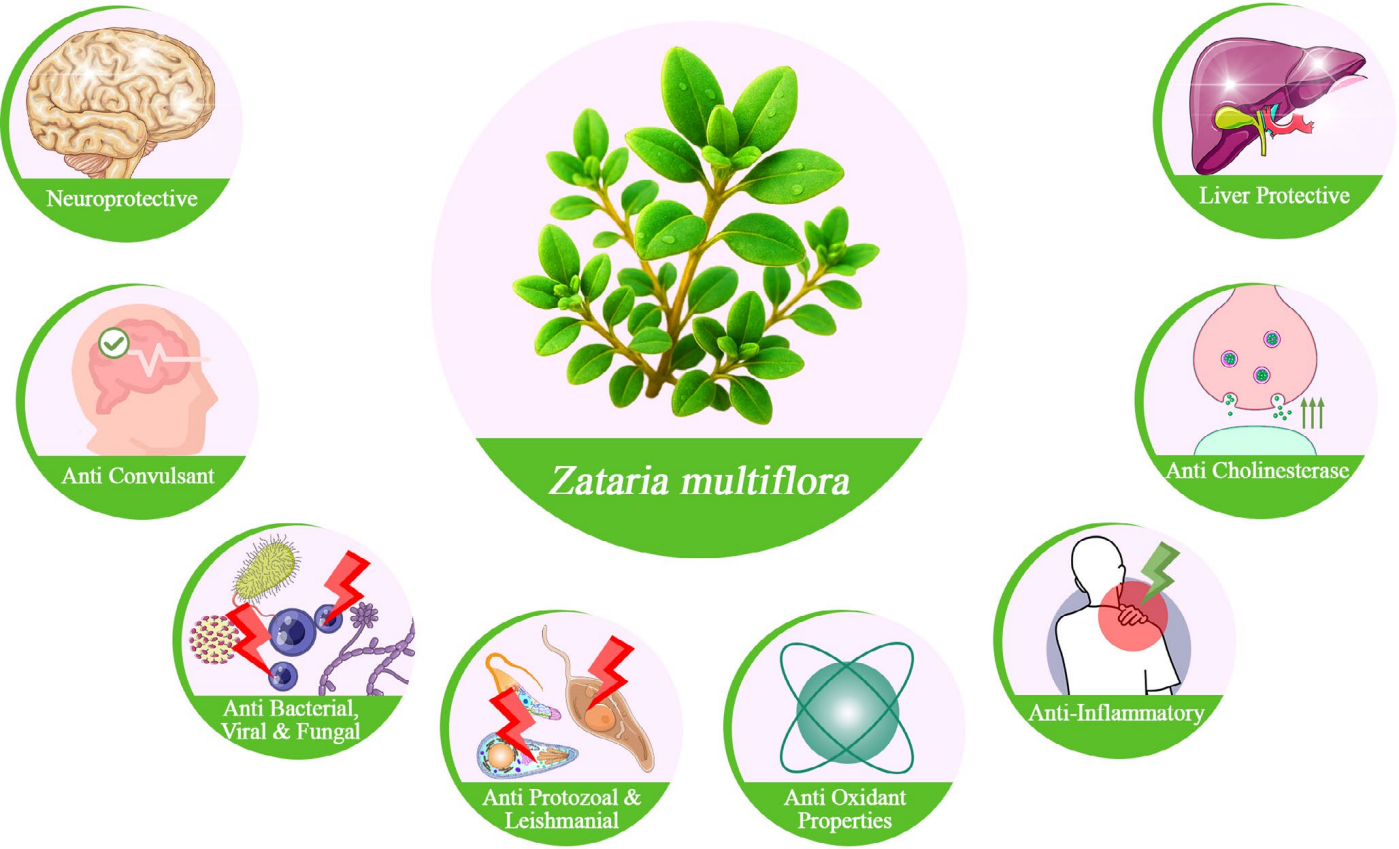
Various biological applications of *Zataria multiflora*.

*Zataria multiflora* (ZM), known as *Avishan‐e‐Shirazi*, is a member of the extensive Lamiaceae family. This aromatic and invaluable plant is exclusively indigenous to the elevated plateaus of Iran, Afghanistan, and Pakistan (Nemati et al. 2015; Nemati and Janitermi 2015). ZM contains several bioactive components, i.e., phenolic compounds, flavonoids, and terpenoids. ZM essential oil (ZMEO) also contains carvacrol and thymol as the main components (Ansari et al. 2025; Golkar et al. 2020; Janitermi and Nemati 2015). This intricate biochemical composition endows ZM with a remarkable and diverse spectrum of significant biological activities (Figure 1), notably including potent antioxidant, broad‐spectrum antimicrobial, anti‐inflammatory, and acetylcholinesterase inhibitory effects (Sharififar et al. 2012; Sharififar et al. 2007; Moradi et al. 2025). This multifaceted collection of pharmacological properties not only makes ZM a valued ingredient in traditional culinary practices but also an intensely fascinating subject for modern pharmaceutical research and development.

Our previous experimental in vitro studies have illustrated the cytotoxic effects of ZM against cell lines of acute promyelocytic leukemia (Baluchi et al. 2018; Asghari et al. 2025), acute lymphoblastic leukemia (Lashkari et al. 2022), multiple myeloma (Anani et al. 2020), and colon carcinoma (Sharififar et al. 2017; Sharififar and Asgharian Rezee 2017). However, the lack of a systematic synthesis of individual attempts has hindered the translation of in vitro findings into pre‐clinical or clinical trials. Therefore, this study aims to systematically review the existing in vitro evidence about the effect of ZM on the growth, metabolism, or proliferation of cancer cells. This review provides a comprehensive approach to highlight the selective cytotoxicity, underlying molecular pathways, as well as the synergistic potential of ZM when combined with traditional anticancer agents. This study focuses on the in vitro studies to facilitate future in vivo or clinical attempts. This study may also aid drug discovery by enhancing ethnobotanical knowledge of a pharmacologically underexplored medicinal plant.

## 2 Methods

This systematic review was conducted in accordance with the Preferred Reporting Items for Systematic Reviews and Meta‐Analyses (PRISMA) guidelines and received approval from the Ethics Committee of Kerman University of Medical Sciences, Kerman, Iran (Ethics Code: IR.KMU.REC.1403.570). The study protocol was not prospectively registered. Our central research question was: “Does *Zataria multiflora* regulate the growth, metabolism, or proliferation of cancer cell lines?”

### 2.1 Search Strategy and Database Selection

A comprehensive search strategy was meticulously developed by a medical librarian (A.S.) employing relevant keywords and their Persian equivalents. These included terms such as *Zataria multiflora*, *Zataria multiflora* Boiss., Cancer, Neoplasm, Tumor, Malignanc*, Carcinoma, and In Vitro. The search spanned major scientific databases, including Web of Science, MEDLINE (via PubMed), Scopus, Embase, and Magiran. We also searched Google Scholar and screened the first 300 records based on relevance ranking, following the pragmatic threshold recommended by Haddaway et al. (2015). The reference lists of all included articles were also screened. The search was executed on September 17, 2025, encompassing all retrieved English and Persian articles published without any temporal restrictions. Table S1 presents the detailed search syntax of each database.

### 2.2 Study Selection

We removed the duplicate records. Subsequently, two independent researchers (A.K. & A.B.) conducted a two‐phase screening process. Initially, retrieved articles were screened by title and abstract to eliminate irrelevant studies. Following this, a comprehensive eligibility assessment was performed on the remaining articles. We included original research papers and experimental studies that investigated the anticancer effects of *Zataria multiflora*, irrespective of its form of administration. Review articles, conference papers, and studies focusing on non‐malignant diseases were excluded. Any disagreements arising during the screening process were resolved through consultation with a third researcher (H.M.V.).

### 2.3 Data Extraction

Data extraction was performed by one author (A.K.), focusing on the following key variables: first author, publication year, country of origin, article language, form of ZM utilized, treated cell line(s), dosage, incubation time(s), IC50 values, experimental methodologies, and primary outcomes. A second reviewer (F.P.) independently cross‐checked all extracted information for accuracy. Discrepancies were adjudicated by a third reviewer (H.M.V.).

#### 2.3.1 Risk of Bias Assessment

The Quality Assessment Tool For In Vitro Studies (QUIN) was employed to assess the quality of included references. QUIN was developed and validated by Sheth et al. (2024) to judge in vitro studies in the field of dentistry. The primary tool consisted of 12 criteria, 4 of which (i.e., “detailed explanation of sample size calculation,” “operator details,” “randomization,” and “blinding”) were excluded due to irrelevance to the cancer cell line‐based experimental models. Two independent authors (A.K. & F.P.) evaluated the evidence for the remaining criteria, including “clearly stated aims/objectives,” “detailed explanation of sampling technique,” “details of comparison group,” “detailed explanation of methodology,” “method of measurement of outcome,” “outcome assessor details,” “statistical analysis,” and “presentation of results.” Discrepancies were resolved by a third reviewer (A.B.). Accordingly, each study was categorized as “low risk,” “moderate risk,” and “high risk.”

## 3 Results

**FIGURE 2:**
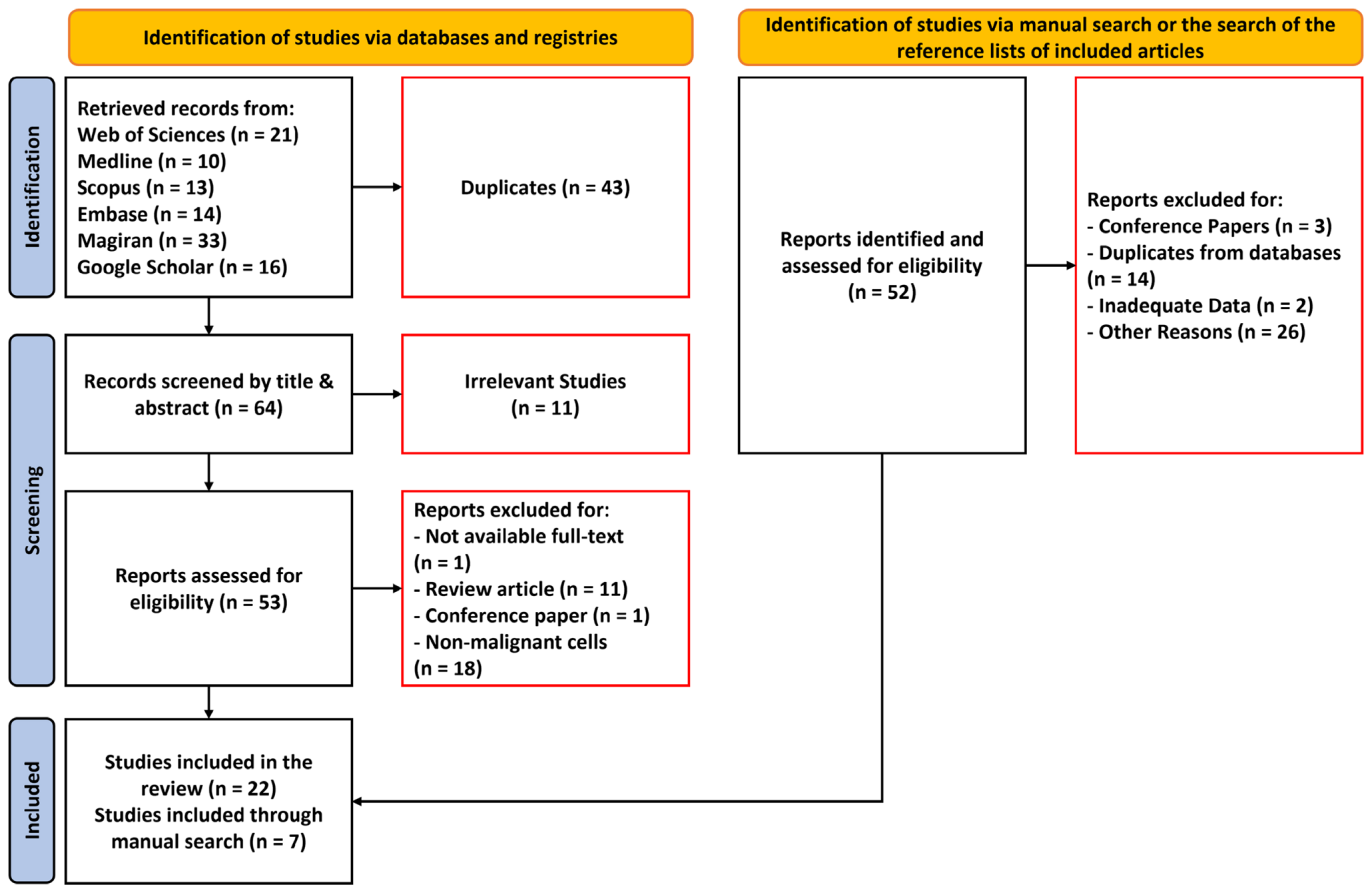
PRISMA flowchart, representing the process of including 29 studies in this systematic review.

The initial systematic search yielded a total of 107 studies. After deduplication, 64 studies proceeded to the title and abstract screening phase. Following title and abstract screening, 53 full‐text articles were assessed for eligibility, 22 of which met the inclusion criteria and were included in the final review. An additional seven studies met the inclusion criteria following a manual search or the search of the reference lists of the initially included papers (Figure 2).

While 3 out of 29 (10.3%) included studies were in Persian (Sabet et al. 2022; Samani et al. 2022; Khorasani et al. 2016), the majority of the articles were in English (*n* = 26, 89.7%). All studies were conducted in Iranian institutions except for the study by Said et al. (2017) from Oman. We found that 24 cell lines, derived from 13 neoplastic diseases, and 6 normal cell types were used to assess the anticancer effects and safety of ZM derivatives. The most studied malignancies were breast cancer (Said et al. 2017; Salehi et al. 2022; Alipanah et al. 2022; Valizadeh et al. 2021; Azadi et al. 2020; Salehi, Behboudi, et al. 2020; Salehi et al. 2017; Ghanbariasad and Osanloo 2020; Mohammadpour et al. 2015; Kavoosi and Rabiei 2015; Salehi, Jamali, et al. 2020), followed by colorectal cancer (Sharififar et al. 2017; Sabet et al. 2022; Samani et al. 2022; Ahani et al. 2020), hematological malignancies (i.e., acute promyelocytic leukemia (Baluchi et al. 2018; Asghari et al. 2025) acute lymphoblastic leukemia (Lashkari et al. 2022), and multiple myeloma (Anani et al. 2020)) hepatocellular carcinoma (Khorasani et al. 2016; Kavoosi and Rabiei 2015; Ghafarkhani et al. 2022; Shokrzadeh et al. 2010), cervical cancer (Azadi et al. 2020; Baharara et al. 2018; Baharara et al. 2016), melanoma (Alipanah et al. 2022; Valizadeh et al. 2021; Osanloo et al. 2024), ovary carcinoma (Mohammadpour et al. 2015; Shokrzadeh et al. 2010), embryonal carcinoma cells (Soltanian et al. 2017), prostate cancer (Zare et al. 2021), glioblastoma (Aghamohammadi et al. 2015), and nasopharyngeal cancer (Kavoosi and Rabiei 2015). Descriptive characteristics of the included studies are detailed in Table 1. Of the 29 references, 17 (58.6%) utilized ZMEO or ZM extract (Table 2), 8 (27.6%) used various nano‐formulations containing ZM (Table 3), and 4 (13.8%) combined both methods (Tables 2 and 3).

**TABLE 1:** Descriptive characteristics of 29 included articles.

References	Language	Form of ZM	Cell line(s) (related cancer, if applicable)	Concentration or dosing of ZM (incubation time)
Asghari et al. (2025)	English	ZM Methanolic Extract	NB4 (Acute Promyelocytic Leukemia) Peripheral Blood Mononuclear Cells	20–80 μg/mL (24, 48 h)
Osanloo et al. (2024)	English	Alginate nanoparticles containing ZMEO	A‐375 (Melanoma) A‐431 (Epidermoid Carcinoma)	39–1250 μg/mL (24 h)
Ghafarkhani et al. (2022)	English	Nanoliposomes containing ZMEO	HepG2 (Hepatocarcinoma)	79.72 μg/mL (48 h)
Salehi et al. (2022)	English	AP‐ZEONE	MCF‐7 (Breast Cancer) MDA‐MB‐231 (Breast Cancer) T47D (Breast Cancer) L929^a^	15.62–500 μg/mL (24, 48, and 72 h)
Sabet et al. (2022)	Persian	ZnO nanoparticles biosynthesized via ZM aquatic extract	HT‐29 (Colon Carcinoma) HEK‐293^b^	31.25–1000 μg/mL (24 h)
Samani et al. (2022)	Persian	ZMEO	HT‐29 (Colon Carcinoma)	1–200 mg/mL (24 h)
Lashkari et al. (2022)	English	ZM methanolic extract	NALM‐6 (Acute Lymphoblastic Leukemia)	20–200 μg/mL (24, 48, 72 h)
Alipanah et al. (2022)	English	Chitosan NPs containing ZMEO	A‐375 (Melanoma) MCF‐7 (Breast Cancer) MDA‐MB‐468 (Breast Cancer)	75–1200 μg/mL (24 h)
Zare et al. (2021)	English	ZMEO	PC3 (Prostate Cancer)	7.5–30 μg/mL (24, 48, 72 h)
Valizadeh et al. (2021)	English	ZMEO ZMSLN	MDA‐MB‐468 (Breast Cancer) A‐375 (Melanoma)	ZMEO: 75–1200 μg/mL (24 h)
				ZMSLN: 75–1200 μg/mL (24 h)
Azadi et al. (2020)	English	ZMEO	4T1 (Breast Cancer) TC1 (Murine Cervical Cancer)	7.8–125 μg/mL (24, 48 h)
Salehi, Behboudi, et al. (2020)	English	ZMEO CS/ZMEONE	MDA‐MB‐231 (Breast Cancer) T47D (Breast Cancer) MCF‐7 (Breast Cancer) L929^a^	ZMEO: 0–250 μg/mL (24, 48, 72 h)
				CS/ZMEONE: 0–500 μg/mL (24, 48, 72 h)
Salehi, Jamali, et al. (2020)	English	ZMEO CP/ZEONE	MDA‐MB‐231 (Breast Cancer) T47D (Breast Cancer) MCF‐7 (Breast Cancer) L929^a^	ZMEO: 0–250 μg/mL (24, 48, 72 h)
				CP/ZEONE: 0–250 μg/mL (24, 48, 72 h)
Ahani et al. (2020)	English	ZMEO	HCT116 (Colon Cancer) SW48 (Colon Cancer)	0–250 ppm (24, 48, 72 h)
Anani et al. (2020)	English	ZM methanolic extract	U266 (Multiple Myeloma)	0–160 μg/mL (24, 48, 72 h)
Ghanbariasad and Osanloo (2020)	English	ZMEO ZM nanoemulsions	MCF‐7 (Breast Cancer) MDA‐MB‐175 (Breast Cancer) MDA‐MB‐231 (Breast Cancer) MDA‐MB‐468 (Breast Cancer)	ZMEO: 0–1280 μg/mL (48 h)
				ZM nanoemulsions: 0–1280 μg/mL (48 h)
Khakzad et al. (2019)	English	ZMEO	K562 (Chronic Myelocytic Leukemia) Peripheral Blood Mononuclear Cells	12.5–100 μg/mL (24, 48, 72 h)
Baluchi et al. (2018)	English	ZM methanolic extract	NB4 (Acute Promyelocytic Leukemia)	0–80 μg/mL (24 h)
Baharara et al. (2018)	English	Silver nanoparticles coated with ZM leaves extract	HeLa (Cervical Cancer)	5–60 μg/mL (24, 48 h)
Sharififar et al. (2017)	English	ZM methanolic extract	HT‐29 (Colon Carcinoma) SW‐48 (Colon Cancer)	0–250 μg/mL (24 h)
Soltanian et al. (2017)	English	ZM methanolic extract	P19 (Embryonal Carcinoma)	0–2560 μg/mL (48, 96, 144 h)
Said et al. (2017)	English	ZM leaves extract	MCAS (Ovarian Cancer) MDA‐MB‐231 (Breast Cancer)	0–1000 μg/mL (24 h)
Salehi et al. (2017)	English	ZMEO, derived from the leaves and flowers of the plant	MDA‐MB‐231 (Breast Cancer) MCF‐7 (Breast Cancer) T47D (Breast Cancer) L929^a^	0–250 μg/mL (48 h)
Khorasani et al. (2016)	Persian	Silver nanoparticles coated with ZM leaves extract	HepG2 (Hepatocarcinoma)	0–60 μg/mL (24, 48 h)
Baharara et al. (2016)	English	Green synthesized gold nanoparticles using ZM leaves	HeLa (Cervical Cancer)	0–400 μg/mL (48 h)
Aghamohammadi et al. (2015)	English	ZM extract	A172 (Glioblastoma) HFFF2^c^	0–200 μg/mL (2 h before radiation)
Mohammadpour et al. (2015)	English	ZMEO	MDA‐MB‐231 (Breast Cancer) SKOV3 (Ovary Carcinoma) HEK^b^	10–200 μg/mL (24 h)
Kavoosi and Rabiei (2015)	English	ZMEO	KB (nasopharyngeal cancer) HepG2 (Hepatocarcinoma) MCF‐7 (epithelial breast cancer)	0–100 μg/mL (24 h)
Shokrzadeh et al. (2010)	English	ZM hydro‐alcoholic extract	HepG2 (Hepatocarcinoma) SKOV3 (Ovary Carcinoma) CHO^d^ Normal Rat Fibroblasts	0–150 μg/mL (48 h)

Abbreviations: AP‐ZEONE, apple‐pectin‐polysorbate‐*Zataria multiflora* essential oil nanoemulsion; CP/ZEONE, citrus‐pectin nano emulsion of *Zataria* essential oil; CS/ZMEONE, chitosan loaded *Zataria multiflora* essential oil nano emulsion; ZM, *Zataria multiflora*; ZMEO, *Zataria multiflora* essential oil.

^a^ Normal murine fibroblast cell line.

^b^ Normal embryonic kidney cells.

^c^ Human Caucasian fetal foreskin fibroblast.

^d^ Normal human ovarian cells.

**TABLE 2:** In vitro cytotoxic effects of different forms of *Zataria multiflora*, excluding nanoformulations.

Form of ZM	Cell line(s)	IC50 (time)	Methods	Main outcome	References
ZM methanolic extract	NB4	Not reported	Trypan Blue Exclusion Assay MTT Assay Annexin V/PI Flowcytometry Quantitative Real‐Time PCR Bioinformatic Analysis Molecular Docking	A synergistic effect was observed for the combination of ZM methanolic extract and ATO, with the best effect noted for 20 μg/mL ZM + 0.25 μM ATO. No cytotoxic effect on PBMCs was reported for 20 μg/mL ZM after 24 h treatment Using different bonding and energy binding mechanisms, thymol and carvacrol can interact with the active site of RAF1.	Asghari et al. (2025)
	PBMC	Not reported			
ZMEO	HT‐29	Not reported	GC/MS Folin–Ciocalteu and Aluminum Chloride Colorimetric Method (to evaluate phenolic and flavonoid content) MTT Assay DPPH free radical scavenging assay ABTS free radical scavenging assay Duncan Test	The free radical inhibition percentages using DPPH and ABTS at a concentration of 1000 ppm were 63.69% and 64.33%, respectively.	Samani et al. (2022)
ZM methanolic extract	NALM‐6	100 μg/mL (48 h)	Trypan Blue Exclusion Assay^a^ MTT Assay Annexin V/PI Flowcytometry Quantitative Real‐Time PCR Molecular Docking	A dose not time‐dependent cytotoxicity Synergistic cytotoxic effect when combined with Doxorubicin No cytotoxic effect for PBMCs Using different bonding and energy binding mechanisms, thymol and carvacrol attach to the residues of the BCL2 and BCL‐xl cavities.	Lashkari et al. (2022)
ZMEO	PC3	26.3 μg/mL (48 h)	MTT Assay^a^ AO/EB staining (Fluorescence Microscopy) Annexin V/PI Flowcytometry Intracellular ROS Detection (Fluorescence Spectroscopy) DNA Fragmentation Assay MMP Cell Cycle Analysis Total Protein Quantification Immunoblotting	Synergistic cytotoxic effect when combined with Doxorubicin Cell cycle arrest in G0/G1 and G2/M phases Increased *Caspase‐3* and *‐9* and *Bax* proteins Decreased *BCL‐2* protein	Zare et al. (2021)
ZMEO	MDA‐MB‐468	380 μg/mL (24 h)	Entrapment Efficacy Assay MTT Assay^a^	Significant reduction in the viability of MDA‐MB‐468 and A‐375 cells treated with *Z. multiflora* essential oil in the form of solid‐lipid nanoparticles	Valizadeh et al. (2021)
	A‐375	59 μg/mL (24 h)			
ZMEO	4T1	Monolayer: 40 ± 3.6, 30 ± 3.0 μg/mL (24, 48 h)	2‐D and 3‐D Cell Culture MTT Assay AO/EB staining (Fluorescence Microscopy) Annexin V/PI Flowcytometry Caspase‐3 Activity Assay In Vivo Experiments	Induction of cytokine secretion in favor of Th1 (i.e., increased TNF‐α, IFN‐γ, and IL‐2, as well as decreased IL‐4) Morphological changes of the cells exposed to ZMEO Apoptosis induction through *Caspase‐3* dependent pathway	Azadi et al. (2020)
		Spheroid: 36 ± 3.0 μg/mL (48 h)			
	TC1	Monolayer: 25 ± 2.6, 22 ± 2.0 μg/mL (24, 48 h)			
		Spheroid: 44 ± 3.5 μg/mL (48 h)			
ZMEO	MDA‐MB‐231	18.3, 29.8, 30.5 μg/mL (24, 48, 72 h)	MTT Assay^a^	Significant proliferation inhibition of both ER‐positive and negative breast cancer cells MDA‐MB‐231 and T47D showed the highest sensitivity in 24 h with decreasing trend by extending the time. MCF‐7 showed the highest sensitivity in 48 h.	Salehi, Behboudi, et al. (2020)
	T47D	7.4, 20.1, 37 μg/mL (24, 48, 72 h)			
	MCF‐7	60.2, 25, 33.1 μg/mL (24, 48, 72 h)			
	L929	Not reported			
ZMEO	MDA‐MB‐231	Monolayer: 18.3, 29.8, 30.5 μg/mL (24, 48, 72 h)	MTT Assay^a^	Significant proliferation inhibition of both ER‐positive and negative breast cancer cells MDA‐MB‐231 and T47D showed the highest sensitivity in 24 h with decreasing trend by extending the time. MCF‐7 showed the highest sensitivity in 48 h.	Salehi, Jamali, et al. (2020)
		Spheroid: 118.4 μg/mL (48 h)			
	T47D	Monolayer: 18.3, 29.8, 30.5 μg/mL (24, 48, 72 h)			
	MCF‐7	Monolayer: 60.2, 25, 33.1 μg/mL (24, 48, 72 h)			
	L929	Not reported			
ZMEO	HCT116	65.02, 50.93, 44.18 ppm (24, 48, 72 h)	MTT Assay^a^ Trypan Blue Exclusion Assay Colony Forming Assay Phase‐contrast microscopy AO/EB staining (Fluorescent Microscopy) Annexin V/PI Flowcytometry Intracellular ROS Detection (Flowcytometry) Quantitative Real‐Time PCR	Decreased expression of *UCP2*, *BCL‐2* Increased expression of *BAX*, *BIK*, *BAK* Suppressed metabolic activity in a dose‐ and time‐dependent manner Lower capacity of exposed malignant cells to establish colonies in a concentration‐dependent manner Morphological changes of the cells exposed to ZMEO Increased cellular ROS level	Ahani et al. (2020)
	SW48	48.64, 42.66, 35.84 ppm (24, 48, 72 h)			
ZM methanolic extract	U266	Not mentioned	Trypan Blue Exclusion Assay MTT assay Antioxidant Activity Assay Quantitative Real‐Time PCR	No significant changes in the expression of *hTERT*, *p21*, and *BCL‐XL* Increased *p53* decreased *c‐myc* No cytotoxic effect for PBMCs. Suppressed metabolic activity in a dose‐dependent manner	Anani et al. (2020)
ZMEO	MCF‐7	76 μg/mL (48 h)	GC–MS DPPH Assay Size Analysis of Nanoemulsions Stability Test of Selected Nanoemulsions MTT Assay^a^	Nano‐emulsion form of *Z. multiflora* essential oil showed superior antioxidant properties and higher toxicity effects against breast cancer cells compared to the non‐formulated form of the essential oil.	Ghanbariasad and Osanloo (2020)
	MDA‐MB‐175	70 μg/mL (48 h)			
	MDA‐MB‐231	104 μg/mL (48 h)			
	MDA‐MB‐468	302 μg/mL (48 h)			
ZMEO	K562	42.82 μg/mL (48 h)	GC–MS Total Phenolic Content FRAP Assay MTT Assay^a^	Although ZMEO was reported as a cytotoxic agent for both cancer cells and normal PBMCs, it was less toxic to normal cells.	Khakzad et al. (2019)
	PBMC	64.97 μg/mL (48 h)			
ZM methanolic extract	NB4	Not mentioned	Trypan Blue Exclusion Assay MTT Assay Quantitative Real‐Time PCR	Decreased *hTERT* Reduced cell viability and metabolic activity in a dose‐dependent manner	Baluchi et al. (2018)
ZM total extract	HT‐29	48.33 ± 26.5 μg/mL (24 h)	MTT Assay^a^	Higher growth inhibitory activity for ZM total extract compared to methanol, chloroform, and petroleum ether fractions.	Sharififar et al. (2017)
	SW‐48	44.22 ± 6.7 μg/mL (24 h)			
ZM leaves extract	P19	262, 299 μg/mL (96, 144 h)	MTT Assay^a^ Trypan Blue Exclusion Assay	The highest cytotoxicity was reported 96 h after exposure, with no further increase with higher incubation time (until 144 h)	Soltanian et al. (2017)
ZMEO, derived from the leaves and flowers of the plant	MCAS MDA‐MB‐231	100 μg/mL	Alamar Blue Staining^a^	The IC50 was noted as “Not Active” since it was calculated to be > 100 μg/mL.	Said et al. (2017)
ZM methanolic extract	MDA‐MB‐231	Spheroid: 118.4 μg/mL (48 h)	GC/MS 2‐D and 3‐D Cell Culture MTT assay AO/EB Fluorescent Staining Annexin V/PI flowcytometry TUNEL Assay DNA Fragmentation Assay Intracellular ROS detection (Flowcytometry) MMP Caspase‐3 Activity Assay DNA Oxidation Analysis Comet Assay Cell Cycle Analysis UV–Vis Spectroscopy Fluorescent Spectroscopic Experiments	Cells in monolayer and spheroid culture demonstrated a decreased dose‐dependent manner. Cell spheroids are less compromised for cell viability Increased Caspase‐3 activity Increased Intracellular ROS level The 8‐oxoguanine assay revealed DNA oxidation and strand break Cell cycle arrest was noted variably due to the dose and time of exposure.	Salehi et al. (2017)
		Monolayer: 29.89 μg/mL (48 h)			
	MCF‐7	25.06 μg/mL (48 h)			
	T47D	20.09 μg/mL (48 h)			
	L929	Not mentioned			
ZM extract	A172 HFFF2	Not mentioned	HPLC MTT Assay PI Fluorescent Staining	Sub‐lethal doses of ZM significantly enhance cancer cell sensitivity to ionizing radiation. The combination of ZM and ionizing irradiation did not demonstrate significant variation, compared to the ionizing radiation alone for normal HFFF2 cells.	Aghamohammadi et al. (2015)
ZMEO	MDA‐MB‐231	141.76 μg/mL (24 h)	Agar Well Diffusion Method (Anti‐Microbial Assay) MTT Assay^a^	Antibacterial and Antifungal effects against *S. aureus* (307 μg/mL), *E. coli* (178 μg/mL), *P. aeruginosa* (95 μg/mL), *S. pyogenes*, *Candida albicans*, and *Trichophyton mentagrophyton*. Lower toxicity for normal cells compared to cancer cells.	Mohammadpour et al. (2015)
	SKOV3	112.2 μg/mL (24 h)			
	HEK	163.2 μg/mL (24 h)			
ZMEO	KB	Six chemotypes of ZMEO were used, all of which demonstrated a strong toxicity across KB, HepG2, and MCF‐7 with 14.5 ≤ IC50 ≤ 19.5 μg/mL (24 h)	GC–MS Total Phenol Content Assay ABTS radical scavenging activity H_2_O_2_ scavenging activity Nitrite scavenging activity MDA scavenging activity MIC MTT Assay^a^	Carvacrol‐rich ZMEO exhibited a higher antibacterial, antifungal, and antitumor effect when compared with thymol‐rich ZMEO.	Kavoosi and Rabiei (2015)
	HepG2				
	MCF‐7				
ZM hydro‐alcoholic extract	HepG2	HepG2: 32.3 ± 2.2 μg/mL (48 h)	Colonogenic Assay^a^	ZM had the highest cytotoxicity for the mentioned cells when compared with *Juniperus sabina* , *Taxus baccata* , and cisplatin.	Shokrzadeh et al. (2010)
	SKOV3	29.6 ± 0.4 μg/mL (48 h)			
	CHO	39.5 ± 1.4 μg/mL (48 h)			
	Normal Fibroblasts	40 ± 2.2 μg/mL (48 h)			

Abbreviations: AO/EB, acridine orange/ethidium bromide staining; AO/PI, acridine orange/propidium iodide staining; AP‐ZEONE, apple‐pectin‐polysorbate‐*Zataria multiflora* essential oil nanoemulsion; ATO, arsenic trioxide; DPPH Assay, diphenylpicrylhydrazyl assay; FRAP, ferric reducing antioxidant power; GC/MS, gas‐chromatography mass spectrometry; MMP, mitochondrial membrane potential; N/A, not applicable; PBMCs, peripheral blood mononuclear cells; SEM, scanning electron microscopy; TEM, transmission electron microscopy; ZM, *Zataria multiflora*; ZMEO, *Zataria multiflora* essential oil.

^a^ The experimental methods utilized for determining IC50.

**TABLE 3:** Cytotoxic effects of nanoformulations synthesized via *Zataria multiflora*.

Nanoformulations	Cell line	IC50 (incubation time)	NP characterization methods	Cytotoxic identification methods	Main outcome	References
Alginate nanoparticles containing ZMEO	A‐375	132 (104–167) μg/mL (24 h)	DLS ATR‐FTIR UV–Vis	MTT Assay*	Encapsulation Efficiency: 86% ± 5% Negligible cytotoxicity and anti‐bacterial effect for carrier material (Alg‐free treatment) Antibacterial effects against *E. coli* (178 μg/mL), *P. aeruginosa* (95 μg/mL), and *S. aureus* (307 μg/mL)	Osanloo et al. (2024)
	A‐431	158 (102–247) μg/mL (24 h)				
Nanoliposomes containing rosemary and ZM.	HepG2	79.72 μg/ml (48 h)	Not reported	MTT Assay* Annexin V/PI Flowcytometry Cell Cycle Analysis	Apoptosis induction Cell cycle arrest in G2 phase	Ghafarkhani et al. (2022)
AP‐ZEONE	MCF7	50.3, 44.54, 32.9 μg/mL (24, 48, 72 h)	PALS DLS FTIR	MTT Assay * AO/EB staining (Fluorescence Microscopy) Annexin V/PI Flowcytometry DNA Fragmentation Assay Comet Assay DNA Oxidation Analysis Intracellular ROS Detection (Flowcytometry) MMP Cell Cycle Analysis DNA Binding Studies	Cytotoxic effect of AP‐ZEONE in a time and concentration‐dependent manner Nanoemulsification enhances the cytotoxic effect of ZM against breast cancer cells Negligible toxicity against normal cells Higher inhibition of multi‐drug resistant triple‐negative breast cancer cell line, compared to ZM alone Morphological changes of the cells exposed to AP‐ZEONE Cell cycle arrest is G2/M phase Interaction of AP‐ZEONE and DNA is through groove binding or partial intercalation.	Salehi et al. (2022)
	MDA‐MB‐231	65.84, 8.68, 1.12 μg/mL (24, 48, 72 h)				
	Epithelial T47D	93.59, 18.69, 1.31 μg/mL (24, 48, 72 h)				
ZnO nanoparticles biosynthesized via ZM aquatic extract	HT‐29	282.4 μg/ml (24 h)	FTIR FE‐SEM XRD EDS MIC	MTT Assay* Quantitative Real‐Time PCR Annexin V/PI Flowcytometry	Increased expression of *P53* and *Casp3* Antibacterial effects against *E. coli* (200 μg/mL), *P. aeruginosa* (200 μg/mL), *B. cereus* (100 μg/mL), and *S. aureus* (100 μg/mL)	Sabet et al. (2022)
	Non‐Malignant HEK‐293	> 282.4 μg/mL (24 h)				
Chitosan NPs containing ZMEO	A‐375	32 (12–84) μg/mL (24 h)	DLS TEM FTIR SEM	MTT Assay*	10%–15% cytotoxicity for free ChiNPs. Dose‐dependent cytotoxicity	Alipanah et al. (2022)
	MCF7	46 (32–67) μg/mL (24 h)				
	MDA‐MB‐468	105 (85–131) μg/mL (24 h)				
Solid lipid NPs containing ZMEO	MDA‐MB‐468	Not calculated precisely	Dynamic Light Scattering	Entrapment Efficacy Assay MTT Assay*	Encapsulation Efficiency: 67% ± 5% Significant reduction in the viability of the free‐SLN‐treated group Significantly higher anticancer activity of ZMSLN compared to ZMEO at all concentrations.	Valizadeh et al. (2021)
	A‐375	Not calculated precisely				
CS/ZMEONE	MDA‐MB‐231	56.9, 52.1, 6.2 μg/mL (24, 48, 72 h)	DLS FTIR	AO/EB staining (Fluorescence Microscopy) MTT Assay* TUNEL Assay DNA Fragmentation FACS Cell Cycle Analysis Intracellular ROS Detection (Flowcytometry) MMP Comet Assay DNA Oxidation Analysis DNA Binding Experiments Circular dichroism (CD) measurements	*Z. multiflora* essential oil incorporated into chitosan nanoparticles suppressed the proliferation of breast cancer cells via inducing apoptosis through a variety of mechanisms (oxidative DNA damage, mitochondrial membrane depolarization, and cell cycle arrest) while sparing normal fibroblasts.	Salehi, Behboudi, et al. (2020)
	T47D	52.7, 1.4, 7.5 μg/mL (24, 48, 72 h)				
	MCF‐7	38.9, 56.2, 21.2 μg/mL (24, 48, 72 h)				
	L929	> 250 μg/mL				
CP/ZEONE	MDA‐MB‐231	Monolayer: 70.92 μg/mL (24 h)	DLS FTIR	MTT* Inverted Microscopy AO/EB staining (Fluorescence Microscopy) DNA Laddering TUNEL Assay Comet Assay Annexin V/PI Flowcytometry Cell Cycle Analysis Intracellular ROS Detection (Flowcytometry) MMP DNA Binding Studies	CP‐loading may increase the stability of ZMEO and sensitize cells toward the treatment. MCF‐7 and T47D are more sensitive to the compound when compared with MDA‐MB‐231. Increased cytotoxicity, mitochondrial instability, and DNA damage/fragmentation following CP/ZEONE exposure. G2/M and S‐phase arrest in monolayer and spheroid culture of MDA‐MB‐231, respectively.	Salehi, Jamali, et al. (2020)
		Spheroid: 65.5 μg/mL (48 h)				
		Monolayer: 62.63 μg/mL (48 h)				
		Monolayer: 20.4 μg/mL (72 h)				
	T47D	Monolayer: 54.8 μg/mL (24 h)				
		Monolayer: 3.31 μg/mL (48 h)				
		Monolayer: 0.0016 μg/mL (72 h)				
	MCF‐7	Monolayer: 33.32 μg/mL (24 h)				
		Monolayer: 68.51 μg/mL (48 h)				
		Monolayer: 5.38 μg/mL (72 h)				
	L929	Not reported				
ZMNE5	MCF‐7 MDA‐MB‐175 MDA‐MB‐231 MDA‐MB‐468	Not reported	Size Analysis of Nanoemulsions Stability Test of Selected Nanoemulsions	GC–MS DPPH Assay MTT Assay*	Increased cytotoxic effects following formulating ZMEO in a nanoemulsion	Ghanbariasad and Osanloo (2020)
Silver nanoparticles coated with ZM leaves extract	HeLa	15, 10 μg/mL (24, 48 h)	DLS UV–Vis Nanoparticle Analysis	TEM MTT Assay* DAPI Staining AO/PI Staining Caspase Activation Assay Annexin V/PI RT‐PCR	Decreased expression of *MMP‐2* Time and dose‐dependent cytotoxicity Smaller ZM‐Ag NPs exhibit a stronger cytotoxicity due to their higher hydrogen peroxide production. Chromatin break was observed through DAPI staining Elevated Caspase‐3 and ‐9 activation	Baharara et al. (2018)
Silver nanoparticles coated with ZM leaves extract	HepG2	40, 30 μg/mL (24, 48 h)	SEM FTIR DLS	MTT Assay* DAPI Staining AO/PI Staining Annexin V/PI (flowcytometry)	Time and dose‐dependent cytotoxicity Chromatin break was observed through DAPI staining	Khorasani et al. (2016)
Green synthesized gold nanoparticles using ZM leaves	HeLa	100 μg/mL (48 h)	UV–Vis Spectroscopy DLS TEM FTIR	MTT Assay Dark Field Microscopy AO/EB Staining DAPI Staining Annexin V/PI Flowcytometry Caspase Activity Assay	Lower toxicity toward normal BMSCs compared to cancer cells. Condensed nuclei and apoptotic bodies were observed through DAPI staining. Elevated Caspase‐3 and ‐9 activation	Baharara et al. (2016)
	BMSC	300 μg/mL (48 h)				

Abbreviations: AO/EB, acridine orange/ethidium bromide; AO/PI, acridine orange/propidium iodide; AP‐ZEONE, apple‐pectin‐polysorbate nanoemulsion of Zataria essential oil; ATR‐FTIR, attenuated total reflectance‐Fourier transform infrared spectroscopy; AP/ZEONE, chitosan nanoemulsion of Zataria essential oil; CP/ZEONE, citrus‐pectin nanoemulsion of Zataria essential oil; DLS, dynamic light scattering; DPPH assay, diphenyl‐picrylhydrazyl assay; EDS, energy‐dispersive X‐ray spectroscopy; FTIR, Fourier transform infrared spectroscopy; GC‐MS, gas chromatography‐mass spectrometry; MMP, mitochondrial membrane potential; NP, nanoparticle; PALS, phase analysis light scattering; ROS, reactive oxygen species; SEM, scanning electron microscopy; TEM, transmission electron microscopy; UV‐VIS, ultraviolet‐visible spectroscopy; XRD, X‐ray diffraction analysis; ZM, Zataria multiflora; ZMEO, Zataria multiflora essential oil.

*The experimental methods utilized for determining IC50.

QUIN was employed to evaluate the quality of the studies. We noted that the included studies were mainly of low to moderate risk of bias, and none of the studies introduced a high risk of bias. The average final score was 72.4%, with 56.3% and 93.8% as the minimum and maximum scores, respectively (Table S2).

## 4 Discussion

Diverse limitations of conventional anticancer therapies have made phytochemicals interesting alternative/complementary treatment options. Botanical derivatives typically contain bioactive compounds, including flavonoids, alkaloids, terpenoids, and phenolic compounds, which further support their anticancer effects (Al Khalily et al. 2025). Despite various preclinical and clinical studies in non‐neoplastic diseases, ZM is relatively unexplored in oncology, with limited data on the safety profile and optimal dosing parameters. Previous in vitro studies on ZM in oncology have primarily focused on cytotoxicity, metabolic activity, and cell cycle alterations. However, systematic reviews of these findings are currently lacking. The distinct nature of various cancer cells, the diverse bioactive compounds of different forms of ZM, and the varying geographical origins of the plant yield conflicting outcomes.

This review demonstrated a significant cytotoxic effect of ZM in almost all investigated cancer cell lines. The IC50 ranges revealed a wide range of sensitivity against ZM derivatives in different cell lines. Cervical, nasopharyngeal, prostate, and hepatocellular cancer cells showed the highest sensitivity, with lower effective concentrations of ZM. Breast cancer, colorectal cancer, melanoma, and hematological malignancies demonstrated an intermediate range of sensitivity, and specific subtypes, such as triple‐negative breast cancer (MDA‐MB‐468) and embryonal carcinoma (P19), demonstrated comparatively higher resistance. The IC50 values were generally reported to be lower when nanoscale ZM formulations were utilized. The cytotoxic effect was also documented for carvacrol and thymol as the main ingredients of ZM. Carvacrol and thymol are also present in oregano ( *Origanum vulgare* ), thyme ( *Thymus vulgaris* ), and ajwain ( *Trachyspermum ammi* ), leading to a common cytotoxic effect for the mentioned plants (Patwa et al. 2025; Balusamy et al. 2018; Al‐Menhali et al. 2015; Karimi et al. 2022; Habib et al. 2021; Seresht et al. 2019).

Our findings indicated that ZM is an effective therapeutic option when combined with conventional cytotoxic agents. Despite the promising efficacy of Doxorubicin, a known DNA‐intercalating anthracycline, it can induce a wide range of adverse effects, including acute nausea and vomiting, stomatitis, alopecia, baldness, hypersensitivity, nail hyperpigmentation, lacrimation, conjunctivitis, immune modulation, neurotoxicity, cardiotoxicity, hepatotoxicity, nephrotoxicity, and bone marrow aplasia (Carvalho et al. 2009; van der Zanden et al. 2021; Ajaykumar 2020). Previous studies have demonstrated a synergistic effect for ZM in combination with Doxorubicin (Lashkari et al. 2022) and Arsenic Trioxide (Asghari et al. 2025). Therefore, ZM is supposed to reduce the need for high‐dose chemotherapy when utilized as a complementary agent. Additionally, the study of Aghamohammadi et al. (2015) revealed that 200 μg/mL of ZM can enhance radiation‐induced apoptosis at doses of 6 and 3 Gy. Due to the damaging effects of radiotherapy on both normal and malignant cells, the administration of ZM may also inhibit the radiotherapy‐induced side effects.

Anticancer agents typically arrest the cell cycle in G1, S, or G2/M phases (Salehi et al. 2022). A significant accumulation of cells in the Sub‐G1 phase is almost always observed after exposure to ZM, confirming its effectiveness in suppressing tumor cell growth and proliferation. Most previous studies have indicated that ZM can also arrest the cell cycle in the G2/M phase in a dose‐ and time‐dependent manner, demonstrating its ability to induce in vitro mitotic arrest and chromosomal instability (Salehi et al. 2022; Salehi, Behboudi, et al. 2020; Salehi et al. 2017; Salehi, Jamali, et al. 2020; Ghafarkhani et al. 2022). However, Zare et al. (2021) reported G1 phase arrest in the PC3 cell line. These discrepancies may be due to cell line‐specific differences or because of time‐ and dose‐dependent effects. According to Salehi et al. (2017), while lower concentrations and shorter exposure to ZM may lead to G1 arrest, higher doses or longer treatments may favor G2/M arrest or apoptosis (Sub‐G1 peak). An S‐phase arrest was also reported in MDA‐MB‐231 cells following ZM exposure in the 3D spheroid cell culture (Salehi et al. 2017; Salehi, Jamali, et al. 2020). This finding highlights the critical role of the growth environment in cellular response to ZM. Although technical challenges have limited spheroid‐based studies, 3D models (which better mimic tumor conditions) should be prioritized over traditional monolayer cultures to explore the unique mechanisms of novel therapeutic agents.

**FIGURE 3:**
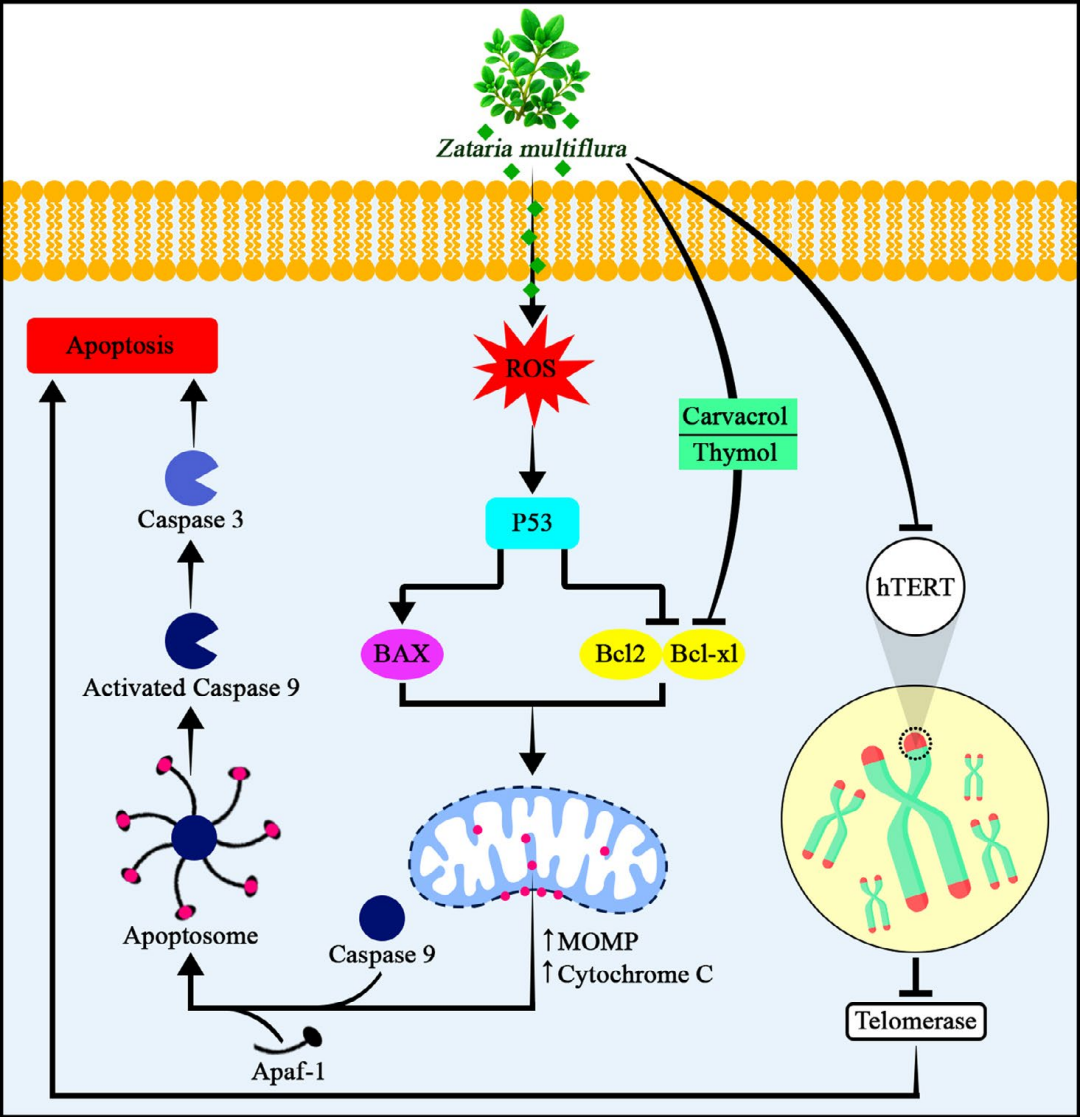
*Zataria multiflora* induces apoptosis in cancer cell lines through ROS generation, anti‐apoptotic protein inhibition, and hTERT suppression.

The gene expression regulatory effects of ZM have been extensively investigated. This role is supported by various bioactive agents in ZM that can influence DNA/RNA interactions with regulatory proteins. Key genes targeted by ZM‐derived bioactive agents mainly regulate tumor cell proliferation, apoptosis, migration, and survival, either directly (through interaction with transcription factors (Lashkari et al. 2022; Anani et al. 2020)) or indirectly (e.g., via miRNAs (Asghari et al. 2025)). Regarding the latter mechanism, Asghari et al. (2025), in a study examining the effects of ZM methanolic extract on the NB4 cell line, found a synergistic effect between ZM and ATO, and observed that this plant extract could induce significant changes in multiple miRNAs involved in tumorigenesis. The anticancer effects of ZM may also involve blocking cancer cell migration by suppressing angiogenesis and extracellular matrix remodulation, evidenced by the downregulation of *VEGF* and *MMP*s (Baharara et al. 2016). A hallmark of cancer cells is their rapid proliferation, which demands mechanisms to preserve DNA integrity during continuous replication. Telomerase is a key enzyme in this process, which ensures DNA integrity by keeping telomeres intact. A study by Baluchi et al. (2018) on a leukemic cell line revealed that ZM methanolic extract suppressed the expression of *hTERT*, the gene encoding the catalytic subunit of telomerase complex. Similarly, Lashkari et al. (2022) observed a reduction in *hTERT* expression in NALM‐6 cells treated with ZM extract. The tumor suppressor *p53*, known for its role in controlling cell cycle progression and apoptosis, is involved in many types of neoplasms. The exposure of cancer cell lines to ZM extract has been reported to induce the expression of *p53*, restricting the proliferation of tumor cells by arresting the cell cycle and promoting apoptosis (Lashkari et al. 2022; Anani et al. 2020; Sabet et al. 2022; Baharara et al. 2016). Supporting this, other regulators of the cell cycle and apoptosis, such as *p21*, were also manipulated by ZM extract. In a study, Lashkari et al. (Lashkari et al. 2022) observed that *p21*, a cyclin‐dependent kinase inhibitor, was suppressed in NALM‐6 cells treated with ZM methanolic extract. Conversely, ZM extract could promote the expression of pro‐apoptotic genes like *BAX*, *BIK*, *BAK*, and *caspase 3* (Sabet et al. 2022; Ahani et al. 2020), while reducing anti‐apoptotic mediators like *BCL‐2* (Ahani et al. 2020). As noted earlier, ZM extract modulates gene expression via interacting with transcription factors, such as *c‐MYC*. Studies by Anani et al. (2020) and Lashkari et al. (2022), examining the effects of ZM methanolic extract on U266 and NALM‐6 leukemic cells, respectively, reported a downregulation of *c‐MYC*. Regarding the crucial role of such transcription factors in regulating oncogenes and tumor suppressors, further studies are warranted to identify other possible functional proteins that ZM ingredients might influence. Additionally, isolating and characterizing bioactive agents from ZM for potential therapeutic use against solid and hematologic cancers is recommended (Figure 3).

Selective toxicity toward cancer cells is crucial in researching new therapeutic agents. IC50 values show that ZM effectively induces apoptosis and inhibits the growth and proliferation of various cancer cells. The IC50 values for ZM against normal cells are almost always higher than those for cancer cells, confirming ZM's lower toxicity toward normal cells. We included ten studies examining the cytotoxic effects of ZM derivatives on normal cells, including L929 (Salehi et al. 2022; Salehi, Behboudi, et al. 2020; Salehi et al. 2017; Salehi, Jamali, et al. 2020), HEK (Sabet et al. 2022; Mohammadpour et al. 2015), peripheral blood mononuclear cells (PBMCs) (Khakzad et al. 2019), HFFF2 (Aghamohammadi et al. 2015), bone marrow mesenchymal stem cells (BMSCs) (Baharara et al. 2016), and CHO (Shokrzadeh et al. 2010). Although ZM is a safe option with known clinical applications outside of cancer treatment, its safety and possible drug interactions in cancer patients still need further investigation.

### 4.1 Limitations

The following limitations should be considered when interpreting the findings of the present study. Due to high heterogeneity in IC50 evaluation methods, exposure times, and intervention formulations, as well as diversity in cancer types and cell lines, quantitative synthesis of dose ranges was not feasible. Instead, we focused on providing critical and mechanistic insights regarding the cytotoxic effects of ZM and potential pathways involved. To facilitate the interpretation of the results, a qualitative comparison of IC50 ranges was provided in the discussion section by cancer type. Further, due to the lack of a sufficiently applicable, standard risk of bias tool for such multidisciplinary studies, QUIN, a quality assessment tool for in vitro studies in dentistry, was employed. Besides, to provide a transparent and unbiased methodology and to mitigate these limitations, we conducted an independent systematic search and screening process and meticulously adhered to the PRISMA guidelines.

Despite these limitations, noteworthy strengths of this study should not be overlooked, including novelty, methodological rigor, comprehensive coverage, critical evaluation, and mechanistic insights.

## 5 Conclusions and Future Perspectives

*Zataria multiflora* is a traditional plant, recognized for its broad spectrum of therapeutic benefits, particularly in managing non‐malignant conditions. While numerous in vitro studies have explored ZM's potential in combating cancer cell lines, in vivo and clinical investigations remain relatively scarce. Due to the high heterogeneity in experimental methods, exposure times, intervention formulations, and cancer cell lines across the included studies, the findings of this study should be interpreted with considerable caution. This review suggests that ZM and its nanoscale derivatives may exert cytotoxic effects across a diverse range of cancer cell lines, including those derived from breast, colorectal, hematological, hepatocellular, cervical, melanoma, ovarian, embryonal, prostate, nasopharyngeal, and glioblastoma cancers. Furthermore, while preliminary data suggest potential synergistic effects with conventional chemotherapeutics, the absence of standardized methodologies and the limited use of physiologically relevant 3D. Further research should prioritize in vivo pharmacokinetic and toxicological studies to refine formulations and determine optimal dosing of ZM in cancer management.

## Author Contributions

**Alireza Khanahmad:** conceptualization, writing – original draft, project administration, data curation. **Ali Bazi:** writing – original draft, investigation. **Fatemeh Peymaninezhad:** writing – original draft, visualization, investigation. **Fariba Sharififar:** investigation, writing – original draft. **Hajar Mardani Valandani:** writing – review and editing, supervision. **Roohollah Mirzaee Khalilabadi:** investigation, writing – review and editing. **Ali Sadatmoosavi:** methodology, writing – original draft.

## Funding

The study was partially funded by the Vice Chancellery of Research and Technology, Kerman University of Medical Sciences (Grant No.: 403000271).

## Ethics Statement

This study was approved by the ethical committee of Kerman University of Medical Sciences (Ethical Code: IR.KMU.REC.1403.570).

## Conflicts of Interest

The authors declare no conflicts of interest.

## Supporting information


**Table S1:** Detailed search strategy for each database.

**Table S2:** QUIN‐based quality assessment of the included articles.

## Data Availability

The authors have nothing to report.
