# Supplementary material for: In Vitro Antineoplastic Effects of Zataria multiflora: A Systematic Review of Mechanisms, Selectivity, and Synergy
Source: Food Sci Nutr. 2026 Jun 30;14(7):e72050. doi: 10.1002/fsn3.72050 (PMC13318923; doi:10.1002/fsn3.72050)
Supplement: Supplementary file 1 — Table S1: Detailed search strategy for each database. [file FSN3-14-e72050-s001.docx]

*Supplementary File 1 - Final Search Syntax*

| Scopus |
| --- |
| ( ( TITLE-ABS ( "Zataria Multiflora" OR "Zataria multiflora Boiss" OR "Z. multiflora" OR "Shirazi thyme" OR "Zataria multiflora Boiss" OR "Zataria bracteata Boiss" OR "Z. multiflora var. elatior Boiss" ) OR AUTHKEY ( "Zataria Multiflora" OR "Zataria multiflora Boiss" OR "Z. multiflora" OR "Shirazi thyme" OR "Zataria multiflora Boiss" OR "Zataria bracteata Boiss" OR "Z. multiflora var. elatior Boiss" ) ) AND ( TITLE-ABS ( cancer* OR neoplasm* OR tumor* OR neoplasia* OR malignanc* OR malignanc* OR carcinoma ) OR AUTHKEY ( cancer* OR neoplasm* OR tumor* OR neoplasia* OR malignanc* OR malignanc* OR carcinoma ) ) ) AND TITLE-ABS ( "In vitro" OR in-vitro ) |
| PubMed |
| (((("Neoplasms"[Mesh]) AND (("Zataria Multiflora"[Text Word] OR "Zataria multiflora Boiss"[Text Word] OR "Z. multiflora"[Text Word] OR "Shirazi thyme"[Text Word] OR "Zataria multiflora Boiss"[Text Word] OR "Zataria bracteata Boiss"[Text Word] OR "Z. multiflora var. elatior Boiss"[Text Word]) OR ("Zataria Multiflora"[Title/Abstract] OR "Zataria multiflora Boiss"[Title/Abstract] OR "Z. multiflora"[Title/Abstract] OR "Shirazi thyme"[Title/Abstract] OR "Zataria multiflora Boiss"[Title/Abstract] OR "Zataria bracteata Boiss"[Title/Abstract] OR "Z. multiflora var. elatior Boiss"[Title/Abstract]))) OR (("Zataria Multiflora"[Title/Abstract] OR "Zataria multiflora Boiss"[Title/Abstract] OR "Z. multiflora"[Title/Abstract] OR "Shirazi thyme"[Title/Abstract] OR "Zataria multiflora Boiss"[Title/Abstract] OR "Zataria bracteata Boiss"[Title/Abstract] OR "Z. multiflora var. elatior Boiss"[Title/Abstract]) AND (Cancer*[Title/Abstract] OR Neoplasm*[Title/Abstract] OR Tumor*[Title/Abstract] OR Neoplasia*[Title/Abstract] OR Malignanc*[Title/Abstract] OR Malignanc*[Title/Abstract] OR Carcinoma[Title/Abstract]))) AND ("In vitro"[Title/Abstract] OR In-vitro[Title/Abstract])) OR ((cancer AND ("Zataria Multiflora" OR "Zataria multiflora Boiss" OR "Z. multiflora" OR "Shirazi thyme" OR "Zataria multiflora Boiss" OR "Zataria bracteata Boiss" OR "Z. multiflora var. elatior Boiss")) AND ("In vitro" OR In-vitro)) |
| Web of Science |
| Topic=(((Zataria NEAR/4 Multiflora) OR (Zataria NEAR/4 multiflora NEAR/4 Boiss) OR (Z. NEAR/4 multiflora) OR (Shirazi NEAR/4 thyme) OR (Zataria NEAR/4 multiflora NEAR/4 Boiss) OR (Zataria NEAR/4 bracteata NEAR/4 Boiss) OR (Z. multiflora var. elatior Boiss)) AND (cancer* OR neoplasm* OR tumor* OR neoplasia* OR malignanc* OR malignanc* OR carcinoma)) AND ("In vitro" OR In-vitro) |
| Embase |
| 1. ((zataria NEAR/4 multiflora):ti) OR ((z. NEAR/4 multiflora):ti) OR ((shirazi NEAR/4 thyme):ti) OR ((zataria NEAR/4 multiflora NEAR/4 boiss):ti) OR ((zataria NEAR/4 bracteata NEAR/4 boiss):ti) OR (z.:ti AND multiflora:ti AND var.:ti AND elatior:ti AND boiss:ti) OR ((zataria NEAR/4 multiflora):kw) OR ((z. NEAR/4 multiflora):kw) OR ((shirazi NEAR/4 thyme):kw) OR ((zataria NEAR/4 multiflora NEAR/4 boiss):kw) OR ((zataria NEAR/4 bracteata NEAR/4 boiss):kw) OR (z.:kw AND multiflora:kw AND var.:kw AND elatior:kw AND boiss:kw) 2. cancer*:ti OR neoplasm*:ti OR tumor*:ti OR neoplasia*:ti OR malignanc*:ti OR carcinoma:ti OR cancer*:kw OR neoplasm*:kw OR tumor*:kw OR neoplasia*:kw OR malignanc*:kw OR carcinoma:kw 3. #1 AND #2 4. #3 AND 'in vitro study'/de 5. 'malignant neoplasm'/exp AND 'zataria multiflora'/exp 6. #5 AND 'in vitro study'/de 7. #4 AND #6 |
| Magiran |
| The core search strategy was structured across three conceptual blocks:  **- Plant and active compounds block:** (“آویشن شیرازی” OR “Zataria multiflora” OR “Z. multiflora” OR “Shirazi thyme”  **- Anticancer/cytotoxic effect block:** (“ضد سرطان” OR “ضدسرطان” OR “ضدتومور” OR “آنتی‌تومور” OR “سمیت سلولی” OR “سایتوتوکسیک” OR “cytotoxic” OR “anticancer”)  **- Study type block (in vitro):** (“برون تن” OR “برون تنی” OR “in vitro” OR “in vitro” OR “آزمایشگاهی”) |
| Google Scholar |
| (("Zataria Multiflora" OR "Zataria multiflora Boiss" OR "Z. multiflora" OR "Shirazi thyme" OR "Zataria multiflora Boiss" OR "Zataria bracteata Boiss" OR "Z. multiflora var. elatior Boiss") AND (cancer* OR neoplasm* OR tumor* OR neoplasia* OR malignanc* OR carcinoma) AND ("In vitro" OR In-vitro)) |
